# Knowledge, Attitudes, and Practices of Undergraduate Dental Students About Sterilization, Disinfection, and Infection Control: A Questionnaire-Based Study

**DOI:** 10.7759/cureus.59525

**Published:** 2024-05-02

**Authors:** Sumit Mohan, Harsh Priyank, Gaurav Kumar, Butta Viswanath

**Affiliations:** 1 Department of Conservative, Endodontics & Aesthetic Dentistry, Dental Institute, Rajendra Institute of Medical Sciences, Ranchi, IND

**Keywords:** interns, undergraduate, disinfection, sterilization, students

## Abstract

Background: The core of infection control in dental settings involves minimizing potential cross-infection risks between patients and from patients to other workers in health care. Infection control is important for promoting enhanced adherence to protocols through sterilization, disinfection, and infection control knowledge, attitudes, and practices (KAP) among undergraduate dental students.

Materials and methods: A cross-sectional survey among 222 undergraduates of Dental Students and Interns of the Dental Institute, Rajendra Institute of Medical Sciences, Ranchi, India, was conducted. KAP of participants related to sterilization and disinfection were assessed before and after educational lectures using a pre-fabricated questionnaire.

Results: All 182 respondents considered the importance of sterilization and disinfection during the dental procedure. While 98.8% had adequate knowledge about isolation and immunization, only 3.8% were vaccinated against hepatitis B virus (HBV). They were perfect in hand hygiene compliance (100%) and awareness regarding autoclave sterilization stood at 78.8%. Mean KAP scores were 7.03 ± 1.39, 10.15 ± 1.40, and 9.

Conclusion: The undergraduate dental students showed a high level of awareness but wide gaps between practice and attitude of sterilization protocols. Therefore, there is a need for interventions that could bridge the theory-practice gap to improve adherence to infection control measures.

## Introduction

Knowledge, attitudes, and practices (KAP) will interact to influence the manner in which people will handle issues or be able to carry out tasks. Indeed, for infection control and patient safety in dental care, the ability to understand must be a key factor for effective measures. Since infections carry great risks that may lead to illnesses and even loss of lives, the implementation of stringent control measures for infections becomes a must within settings to reduce such risks [1].

Infection control, therefore, is a whole range of measures whose intention is to cut down the transfer of infectious agents between the patient and healthcare workers. Ignoring these exposes the involved practitioners not only to compromising patient safety but also to occupational hazards. In the dental set-up, where there are so many routes of infection transmission, strict observance of the protocols on sterilization and disinfection assumes great importance. However, the concern related to the adequacy of infection control practice is especially the dental students. Recent scrutiny is upon the knowledge and application of correct sterilization procedures by dental students [2,3].

Indeed, in recent years, the focus on infections has become more intense against the background of growing awareness of infectious diseases and their global impact on infection control measures in all disciplines of health care. Within dental education, there arises a need to inculcate clearly understood protocols relevant to sterilization, disinfection, and infection control paramount to patient and practitioner safety and well-being. Improved knowledge and infection control practices had been made in their place; however, some knowledge gaps still existed, more so among dental students [4-6]. Fixing these gaps is essential not only to secure the health of the patient but also to inculcate lifelong habits of meticulous infection control practices among dental professionals of the future.

The complexity of dental procedures, together with the potential for cross-infection by a number of routes, requires an all-inclusive education approach to the control of infection. This extends to the theoretical knowledge of the same into the building up of positive attitudes for the measures of infection control and putting this understanding into evidence-based practice. More to that, for education interventions to be successful, they should take into account the institutional policies and the sociocultural factors influencing the behaviors of students, with regard to resource limitations [7]. Dental education institutes have the most paramount responsibility of bridging the gap between theoretical knowledge and its practical application to mold a whole new breed of dental practitioners conscious of patient safety and the strictest standards of infection control.

Considering the critical importance of infection control in dental settings and possible fallouts emanating from lapses in adherence to the protocols, it becomes incumbent to assess the KAP of students regarding sterilization, disinfection, and infection control. This research thus seeks to assess the epidemiologic and cross-sectional dimensions among undergraduate dental students studying in the Dental Institute, Rajendra Institute of Medical Sciences, Ranchi, India, to identify areas that may require further inputs to strengthen educational interventions on best practices of infection control. These findings will be of informative value to target interventions and curriculum enhancements when fostering a culture of rigorous infection control in dental education and practice.

## Materials and methods

The research was conducted at the Dental Institute of the Rajendra Institute of Medical Sciences, Ranchi, India. It was aimed at assessing the KAP of sterilization and disinfection among undergraduate dental students and interns. The research was conducted with ethical clearance from the Institutional Ethics Committee of Rajendra Institute of Medical Sciences (IEC/RIMS/2020/47) for the genuineness of the research process. A total of 222 participants were considered in the research, including undergraduate dental students and interns belonging to the same institution. The sampling method that was used was convenient sampling. This was used for reaching out to willing members who are easily accessible and within the academic institute.

The inclusion criteria were structured in a way that would ensure the participants fell within the study objectives. The participants also had to give voluntary consent, considering this was a major part of the ethical aspect of the research study. Exclusion criteria were used to sustain consistency in the baseline knowledge between participants. The exclusion criteria were that a history of expertise beyond that provided in educational lectures or medical illnesses would compromise the patient's ability to understand the material.

The research followed the approach of collecting data concerning participants' KAP of sterilization and disinfection using a cross-sectional questionnaire. The participants were educated during two 45-minute lectures given the week before administering the questionnaire. The lecturers were geared toward empowering the students with concepts of sterilization, disinfection, and infection control in dental practice for quality responses during later evaluation [6-9].

Educational sessions ended by giving them a prefabricated questionnaire for completion. The questionnaire used, most likely from previous researchers, had questions relating to the knowledge of participants on protocols of sterilization, their attitude toward measures to control infection, and practices within the reported clinical settings. This called for the institution of a one-week cooling-off period before the respondents would answer the questionnaire to leave room for time to reflect on the educational content.

## Results

The details of the respondents are shown in Table 1.

**Table 1:** Details of respondents n: number of participants; %: percentage

Total students N = 222		
Students participated in the study n = 182		
BATCH	BOYS, n (%)	GIRLS, n (%)
BDS I Year	18 (32.14%)	38 (67.85%)
BDS II Year	4 (8.1%)	37 (91.9%)
BDS III Year	10 (7.5%)	36 (92.5%)
BDS IV Year	4 (34%)	20 (66%)
Interns	2 (40%)	13 (60%)

Results of the study indicated that all 182 participants believed that sterilization and disinfection were necessary during dental procedures. Although 180 participants (98.8%) had sufficient knowledge regarding the importance of isolation and immunization only 7 students (3.8%) had been vaccinated against hepatitis B virus (HBV). Results of our study showed that 91.3% of students (166) sanitized the dental chairs, X-ray machines, and other clinical operative equipment before use while 111 students (61.3%) performed oral rinses to patients before any procedure. The majority of the students 144 (78.8%) acknowledged autoclave as an accepted method of sterilization. The responses to the structured questionnaire are summarized in Table 2.

**Table 2:** Questionnaire for the study n: number of participants; %: percentage

S. No.	Question	Responses	N	Frequency
1	Do you wash your hands before and after treating/examining a patient	Yes	182	100%
		No	0	0%
2	What do you use for washing your hand in a dental operatory	Antiseptic solution	80	43.8%
		Plain soap	9	5%
		Handwash	93	51.3%
3	Are you aware about the importance of isolation of the operating field in infection control	Yes	180	98.8%
		No	2	1.3%
4	Have you take vaccine against hepatitis B	Yes	7	3.8%
		No	175	96.2%
5	Is tetanus vaccine essential in case of prick injury	Yes	39	21.3%
		No	143	78.7%
6	Do you prefer to give an pre procedural mouth rinse	Yes	111	61.3%
		No	71	38.8%
7	Which disease has the highest spread rate through saliva	Hepatitis B	100	55%
		AIDS	23	12.5%
		TB	48	26.3%
		All of the above	0	0.0%
		None	11	6.3%
8	Which is the best method of sterilization	Autoclave	144	78.8%
		Boiler	2	1.3%
		Surface Disinfection	36	20.0%
9	Can improper sterilization lead to cross infection	Yes	182	100%
		No	0	0%
10	Is it necessary to disinfect dental chair, x ray, clinic and operatory	Yes	166	91.3%
		No	9	5%
		Cant Say	7	3.8%

In our study, the average scores of KAP were 7.03 + 1.39, 10.15 + 1.40, and 9.59 + 1.32, respectively. The groups were found to be significantly different from each other (≤0.01). Knowledge and practice values showed a significant association (p ≤ 0.01); however, there were no significant differences between knowledge and attitude or attitude and practice (Table 3).

**Table 3:** Mean (SD) of knowledge, attitudes, and practices (KAP) regarding infection control SD: standard deviation p-value was considered significant if <0.05.

Batch	Knowledge mean ± SD	Attitude mean ± SD	Practice mean ± SD
BDS I Year	6.96 ± 1.35	10.38 ±1.23	9.80 ± 1.46
BDS II Year	6.44 ± 1.62	10.10 ± 1.54	9.28 ± 1.47
BDS III Year	6.98 ± 1.36	10.16 ± 1.42	9.66 ± 1.38
BDS IV Year	7.08 ± 1.26	10.28 ± 1.31	9.57 ± 0.92
Interns	7.69 ± 1.36	9.86 ± 1.52	9.66 ± 1.38
Total	7.03 ± 1.39	10.15 ± 1.40	9.59 ± 1.32
p-value	0.000	0.387	0.022

## Discussion

The present study aimed to evaluate the KAP of undergraduate dental students at Rajendra Institute of Medical Sciences, Ranchi, India, concerning sterilization, disinfection, and infection control. Notably, the study found a high level of awareness (100%) among participants regarding sterilization and disinfection, with the majority (95.5%) acknowledging the importance of hand hygiene. However, there were gaps in specific areas, as only 78.8% of students were familiar with autoclaves. These findings align with previous research [9,10], suggesting a consistent trend in awareness levels across different study populations.

Interestingly, despite awareness of the risks associated with diseases such as hepatitis B, only a small percentage (3.8%) of participants were vaccinated against it. Similarly, awareness of the transmission routes of AIDS and tuberculosis was relatively low (12% and 26.3%, respectively), contrary to existing literature [11]. These disparities highlight potential areas for improvement in educational interventions targeting infection control practices among dental students.

The study's inference that students possess adequate knowledge but lack in attitude and practice underscores the importance of bridging the gap between theoretical understanding and practical application. This disparity may be attributed to discrepancies between students' professed intentions and their actual behavior in clinical settings [12]. To address this, educational efforts should focus on imparting not only knowledge but also fostering positive attitudes toward infection control measures. Incorporating dedicated academic sessions and lectures on infection control, including the significance of immunization against hepatitis B and precautions against HIV and TB, can help instill a culture of adherence among students [13-15].

However, the study's limitations must be acknowledged. The sample size may not fully represent the entire population of undergraduate dental students, potentially limiting the generalizability of the findings. Additionally, self-reporting bias may have influenced participants' responses, leading to potential overestimation of understanding or adherence to protocols. Moreover, the reliance on survey-based data might have restricted the depth of understanding of students' actual clinical practices. Future research should consider exploring factors such as resource availability, institutional policies, and cultural influences on adherence to infection control protocols. Furthermore, investigating the perspectives of senior students and faculty members could provide a more comprehensive understanding of the dynamics shaping infection control practices in dental education and practice. Addressing these limitations will contribute to enhancing infection control measures within dental education and practice contexts.

## Conclusions

According to the study's limitations, undergraduate dentistry students at Rajendra Institute of Medical Sciences in Ranchi, India, reported a high level of compliance with infection control measures. Through classroom instruction and continuing professional education, faculty might further encourage students to follow infection control protocols. It was also noted that students had a good amount of understanding about sterilization and infection control methods, but that this information had to be put into practice every day.

## References

[1] Jain M, Sawla L, Mathur A, Nihlani T, Ayair U, Prabu D, Kulkarni S (2010). Knowledge, attitude and practice towards droplet and airborne isolation precautions amongs dental health care professionals in India. Med Oral Patol Oral Cir Bucal.

[2] Harte JA (2004). Looking inside the 2003 CDC dental infection control guidelines. J Calif Dent Assoc.

[3] Singh A, Purohit BM, Bhambal A, Saxena S, Singh A, Gupta A (2011). Knowledge, attitudes, and practice regarding infection control measures among dental students in Central India. J Dent Educ.

[4] Laheij AM, Kistler JO, Belibasakis GN, Välimaa H, de Soet JJ (2012). Healthcare-associated viral and bacterial infections in dentistry. J Oral Microbiol.

[5] Ayatollahi J, Ayatollahi F, Ardekani AM, Bahrololoomi R, Ayatollahi J, Ayatollahi A, Owlia MB (2012). Occupational hazards to dental staff. Dent Res J (Isfahan).

[6] Cheng HC, Su CY, Huang CF, Chuang CY (2012). Changes in compliance with recommended infection control practices and affecting factors among dentists in Taiwan. J Dent Educ.

[7] Abreu MH, Lopes-Terra MC, Braz LF, Rímulo AL, Paiva SM, Pordeus IA (2009). Attitudes and behavior of dental students concerning infection control rules: a study with a 10-year interval. Braz Dent J.

[8] Thomas MV, Jarboe G, Frazer RQ (2008). Infection control in the dental office. Dent Clin North Am.

[9] Sukhlecha AG, Vaya S, Parmar GG, Chavda KD (2015). Knowledge, attitude, and practice regarding sterilization among health-care staff in a tertiary hospital of western India. Int J Med Sci Public Health.

[10] Kamate SK, Sharma S, Thakar S (2020). Assessing knowledge, attitudes and practices of dental practitioners regarding the COVID-19 pandemic: a multinational study. Dent Med Probl.

[11] Sofola OO, Savage KO (2003). Assessment of the compliance of Nigerian dentists with infection control: a preliminary study. Infect Control Hosp Epidemiol.

[12] Ogden GR, Bahrami M, Sivarajasingam V, Phillips G (1997). Dental students’ knowledge and compliance in cross infection control procedures at a UK Dental Hospital. Oral Dis.

[13] de Souza RA, Namen FM, Galan J Jr, Vieira C, Sedano HO (2006). Infection control measures among senior dental students in Rio de Janeiro State, Brazil. J Public Health Dent.

[14] Myers JE, Myers R, Wheat ME, Yin MT (2012). Dental students and bloodborne pathogens: occupational exposures, knowledge, and attitudes. J Dent Educ.

[15] Alavian SM, Mahboobi N, Mahboobi N, Savadrudbari MM, Azar PS, Daneshvar S (2011). Iranian dental students' knowledge of hepatitis B virus infection and its control practices. J Dent Educ.

